# Protein Characterization of a Candidate Mechanism SNP for Crohn's Disease: The Macrophage Stimulating Protein R689C Substitution

**DOI:** 10.1371/journal.pone.0027269

**Published:** 2011-11-07

**Authors:** Natalia Gorlatova, Kinlin Chao, Lipika R. Pal, Rawan Hanna Araj, Andrey Galkin, Illarion Turko, John Moult, Osnat Herzberg

**Affiliations:** 1 Institute for Bioscience and Biotechnology Research, University of Maryland, Rockville, Maryland, United States of America; 2 National Institute for Standards and Technology, Gaithersburg, Maryland, United States of America; 3 Department of Cell Biology and Molecular Genetics, University of Maryland, College Park, Maryland, United States of America; 4 Department of Chemistry and Biochemistry, University of Maryland, College Park, Maryland, United States of America; Griffith University, Australia

## Abstract

High throughput genome wide associations studies (GWAS) are now identifying a large number of genome loci related to risk of common human disease. Each such locus presents a challenge in identifying the relevant underlying mechanism. Here we report the experimental characterization of a proposed causal single nucleotide polymorphism (SNP) in a locus related to risk of Crohn's disease and ulcerative colitis. The SNP lies in the MST1 gene encoding Macrophage Stimulating Protein (MSP), and results in an R689C amino acid substitution within the β-chain of MSP (MSPβ). MSP binding to the RON receptor tyrosine kinase activates signaling pathways involved in the inflammatory response. We have purified wild-type and mutant MSPβ proteins and compared biochemical and biophysical properties that might impact the MSP/RON signaling pathway. Surface plasmon resonance (SPR) binding studies showed that MSPβ R689C affinity to RON is approximately 10-fold lower than that of the wild-type MSPβ and differential scanning fluorimetry (DSF) showed that the thermal stability of the mutant MSPβ was slightly lower than that of wild-type MSPβ, by 1.6 K. The substitution was found not to impair the specific Arg483-Val484 peptide bond cleavage by matriptase-1, required for MSP activation, and mass spectrometry of tryptic fragments of the mutated protein showed that the free thiol introduced by the R689C mutation did not form an aberrant disulfide bond. Together, the studies indicate that the missense SNP impairs MSP function by reducing its affinity to RON and perhaps through a secondary effect on *in vivo* concentration arising from reduced thermodynamic stability, resulting in down-regulation of the MSP/RON signaling pathway.

## Introduction

Until recently, information on which variants within the human genome contribute to increased risk of common human disease was fragmentary and often statistically weak. New chip based technologies are now providing relatively unbiased and reliable information on which single nucleotide polymorphisms (SNPs) are significantly associated with altered risk for a number of common diseases. The current generation of GWAS typically includes several thousand individuals with the disease of interest and a similar number of control individuals without the disease (for example [1]). These studies, and more recently, meta-studies combining data from a number of individual experiments [2], [3] have already led to identification of up to a 100 risk associated loci for some individual diseases. These new data open up the possibility of a deeper understanding of the nature of complex trait disease and consequential advances in treatment, diagnosis, prognosis and prophylaxis. Identification of the mechanism underlying each risk locus is non-trivial, since a typical microarray contains complementary oligos for only 500,000 to a million common SNPs (those with a population allele frequency greater than 1%), while the number of known common SNPs is more than 10 times this (http://www.ncbi.nlm.nih.gov/projects/SNP/). It is thus unlikely that a sampled (tag) SNP found to be associated with altered disease risk (a ‘marker’ SNP) is in fact playing a direct role in the disease mechanism. Rather, because of incomplete recombination within the population, the presence of a marker SNP will be correlated with the presence of the genetic variant actually affecting *in vivo* function, through linkage disequilibrium (LD). For any given marker associated with increased disease risk, each of the SNPs in LD is a candidate for involvement in the disease mechanism. The range of LD varies widely across the genome, and thus the number of candidate SNPs does also, from approximately 100 to of the order of 10,000 in the highly linked MHC region of chromosome 6 (Pal and Moult, unpublished).

It is likely that, in each locus, no more than one of the many candidate SNPs is involved in disease mechanism. It is also possible that in any given locus none of the identified candidate SNPs is the mechanism for a number of reasons. A mechanism SNP may directly influence phenotype and hence disease risk through a number of known processes, such as amino acid substitutions altering *in vivo* protein function, altered mRNA level via effects on transcription factor binding, microRNA action, message half-life or splicing, and changed translational efficiency through effects on mRNA structure and codon efficiency. Although most disease related loci encompass one or more genes, some are in so-called gene deserts, suggesting additional mechanisms may exist. At present, the relevance of each mechanism is unknown. Also unknown is the distribution of impact levels on protein function – how common are subtle changes in gene function versus major changes? The best understood mechanisms are those arising from non-synonymous variants (missense and nonsense single base variants, changing an amino acid in a coding region or truncating the polypeptide chain), which may alter *in vivo* activity of the affected protein by effects on folding and thermodynamic stability, ligand and partner binding, catalysis, allosteric regulation, and post-translational modification. In previous work, we have developed two machine-learning methods for identifying which missense variants have a high impact on protein function. One of these methods makes use of a set of features of protein three-dimensional structure to identify those variants that significantly decrease protein thermodynamic stability, and we have shown that this is the major mechanism in monogenic disease [4]. The second method makes use of the extent of local sequence conservation and the nature of an amino acid substitution to identify variants that affect *in vivo* function by any missense mechanism [5]. The methods have been validated by analysis of a set of variants causative of the monogenic disease phenylketonuria (Shi, Sellers & Moult, In press) and a set of common SNPs that have been investigated experimentally [6]. Application of these methods to missense SNPs in the human genome has shown that approximately a quarter have high impact on protein function *in vivo*, with decrease in protein thermodynamic stability again playing the major role [5]. The high incidence of these high impact SNPs suggests they are likely to play a significant role in common disease. To investigate this possibility, we have now applied the methods to GWAS results from a Wellcome Trust Case Control Consortium (WTCCC) study of seven common diseases [1] and follow-up studies. The analyses show that about one third of all loci have at least one missense SNP predicted to have a large impact on protein function, and these represent possible molecular mechanisms for those loci. These results support a major role for high impact non-synonymous SNPs.

Here we present an experimental study of possible molecular mechanisms for a predicted high impact missense SNP in macrophage stimulating protein (MSP, also known as hepatocyte growth factor like protein (HGF-like), associated with a disease related locus for Crohn's disease on chromosome 3. This SNP has earlier been identified as associated with inflammatory bowel disease in a more focused gene centric study UC [7]. Recently, the same SNP was reported as associated with the chronic liver disease primary sclerosing cholangitis, a disease that affects 2.4–7.5% of individuals with inflammatory bowel disease [8].

Crohn's disease (CD) and ulcerative colitis (UC) are chronic inflammatory bowel diseases, with a genetic component, and a relatively high heritability of 50–60% [9]. A series of GWAS and meta GWAS have so far identified 71 and 47 susceptibility loci for CD and UC, respectively [2], [10]. Genes in these loci span widely distributed functions, but a number are involved in the inflammatory response and in particular in autophagy. As described later, analysis of the locus on chromosome 3, first identified in a GWA study [1] identifies a single high impact non-synonymous SNPs in the MST1 gene, resulting in substitution of Arg689 by Cys in the MSP β chain.

MSP is a component of a signaling pathway that regulates aspects of the innate immune response to infection and cellular stress. MSP and its receptor, RON receptor tyrosine kinase, are involved in macrophage chemotaxis and activation. Macrophage function in normal cell proliferation, adhesion, motility and apoptosis is well documented, and their activity is elevated in many solid tumors. Such elevated activity correlates with metastasis and poor cancer prognosis. Hence, the MSP/RON signaling pathways have been proposed as a potential cancer therapeutic target [11].

MSP is a serum protein that circulates in the blood as an inactive single-chain precursor (pro-MSP) comprising two chains, α and β [12]. Cleavage of the Arg483-Val484 peptide bond yields the mature active MSP α/β heterodimer, linked by a disulfide bond between Cys468 and Cys588. The α chain (MSPα) contains four kringle domains and the β chain (MSPβ) adopts a trypsin-like serine protease fold, but lacks the catalytic triad and proteolytic activity. Matriptase-1, a type II transmembrane serine protease expressed on epithelial cells and macrophages, has been shown to specifically cleave and activate pro-MSP [13].

The mature form of RON contains two chains (α and β) linked by a disulfide bond. The α chain is entirely extracellular and encodes for the N-terminal half of a Sema domain, a domain unique to the RON/MET subfamily of receptor tyrosine kinases. The β chain comprises an extracellular polypeptide region that includes the C-terminal half of the Sema domain, a PSI domain (acronym for plexin, semaphorins, integrins) and four immunoglobulin-like domains, IPT_1_ through IPT_4_. A single transmembrane segment followed by an intracellular tyrosine kinase domain complete the β chain. A gene-focused study has found two non-synonymous SNPs (R523Q and G1335R) of RON to be associated with increased risk of CD and UC [14].

Several studies established that MSPβ binds to the RON Sema domain with high affinity [15], [16], [17], [18]. Competitive ELISA experiments showed that MSPα binds weakly to RON Sema-PSI [18] whereas competition experiments with ^125^I radiolabeled MSP indicated that MSPα did not bind to RON in intact cells [17] and co-immunoprecipitation assays failed to detect MSPα binding to a RON construct that contained the Sema-PSI and a portion of the ensuing IPT_1_ [19]. By comparison, the α and β chains of the MSP homolog, HGF, exhibit a reverse trend in the binding affinities to the HGF receptor, MET, which is a homolog of RON [20]. A proposed 3-D model based on a combined small-angle X-ray scattering and cryo electron tomography study suggested the interactions of both the HGF α and β chains with the MET Sema domain [21]. By analogy, it is possible that both α and β chains of MSP interact with RON Sema even though the binding of MSPα is weak.

## Results and Discussion

### Candidate SNP analysis

The initial WTCCC GWA Study [1] identified nine loci where tag SNPs are significantly associated with increased risk of Crohn's disease. One of these is on chromosome 3, and contains three marker SNPs. Two of these markers are in introns in bassoon (presynaptic cytomatrix protein, BSN, and the third is a synonymous SNP in that gene. The linkage disequilibrium region for chromosome 3 locus, in 3p21, spans 20 genes, an unusually high number. Penetrance analysis finds a total of 148 candidate SNPs, seven of which are non-synonymous (Table 1).

**Table 1:** Candidate non-synonymous mechanism SNPs in the Chromosome 3 locus associated with increased risk of Crohn's disease.

SNP ID	Gene ID	Substitution	Profile Impact^a^	Stability Impact^a^
rs34762726	BSN	A741T	NA	NA
rs2005557	BSN	T3863A	+0.59	NA
rs13068038	CCDC36	D430E	+1.79	NA
rs34823813	RNF123	R854H	+1.99	NA
rs13072748	VLLR9392	R5K	+1.77	NA
rs13077498	C3ORF62	E110K	+2.90	NA
rs3197999	MST1	R689C	−1.13	+0.52

a Profile and stability impact scores are the outputs from the sequence profile and structure stability support vector machines respectively. Positive scores reflect an assignment of a low or negligible impact on *in vivo* protein function, negative scores an assignment of high impact on protein function. The higher the absolute score, the higher the confidence of the assignment. Scores >|0.5| are considered high confidence. NA indicates there is inadequate sequence or structure information available to make an assignment. (See Methods and [5] for details). Only the SNP in MST1 is assigned a high impact on protein function.

All but two of the non-synonymous SNPs have high confidence profile based assignments of a low impact on *in vivo* function. No assignment is available for the A741T in BSN, but this gene is an unlikely candidate on biological grounds, since it is involved in neuronal development, and only expressed in brain. The single high confidence high impact assignment is for rs3197999 in MST1, resulting in the substitution of an arginine by a cysteine, at position 689 of MSP. This SNP is in almost complete LD with the marker (R^2^ = 0.95). The sequence profile analysis classifies this substitution as high impact on protein function, with a high confidence support vector machine (SVM) score (−1.13), primarily determined by the facts that close orthologs all have an arginine at this position and no homolog has a cysteine. In contrast, the structure method classifies the change as benign (high confidence SVM score of 0.52). Thus the initial computational analysis suggests this SNP is the only known high impact non-synonymous candidate in the chromosome 3 locus, and impacts the function of MSP *in vivo* through some mechanism unrelated to protein stability. The MSP R689C substitution lies on the surface of the MSP β-chain [22]. The computational results may reflect a role for this residue in a number of processes that could be disrupted by the amino acid substitution and so contribute to Crohn's disease susceptibility. Principle among these are a reduction in binding affinity to RON Sema, structural perturbation resulting from an incorrect disulfide bond formed with the newly introduced cysteine, or impaired activation of pro-MSP by matriptase-1. To examine the contributions of these effects, we undertook to characterize the MSPβ and its interaction with RON extra cellular domain constructs *in vitro* using well-purified proteins. Although the computational analysis classified the substitution as not affecting protein stability, and the surface location supported that analysis, we nevertheless also measured the change in protein stability resulting from the substitution to directly determine any contribution from this factor.

### Thermostability of MSPβ

**Figure 1:**
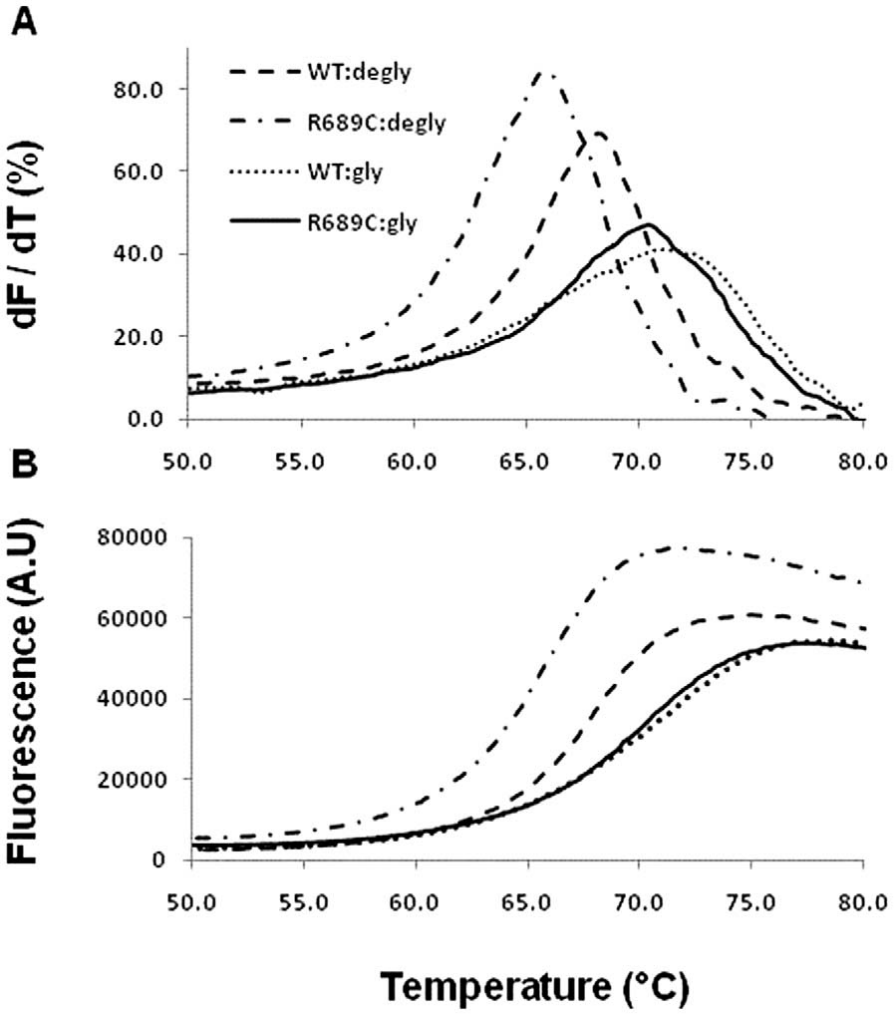
MSPβ thermal stability monitored by DSF. Four representative experiments are shown, one for each MSPβ: Wild-type glycosylated, wild-type deglycosylated, glycosylated R689C, and deglycosylated R689C. The melting curves were measured by heating the samples at a rate of 2°C per minute (B). The T_m_ values were calculated as the maximum of the first derivative of the melting curves (A). The first derivative values (dF/dT) are normalized and given in %, where 100% corresponds to the highest peak of the entire experiment set. The fluorescence values are on an arbitrary scale (AU). Table 2 provides the T_m_ values. The R689C variant melts at approximately 2.5 K lower temperature than the wild type for the deglycosylated protein and 1.6 K lower for the glycosylated form.

DSF is a useful technique for rapid assessment of protein thermal stability. With this approach, the proteins are subjected to gradually increasing temperature and the difference between the melting temperatures (T_m_) of two samples being compared is measured using a fluorescent dye (SYPRO Orange was used in the current study) whose emission properties change upon interaction with unfolded protein. MSPβ has a single glycosylation site and was produced in *Drosophilae melanogaster* Schneider 2 (S2) cells, which may yield a different glycosylation pattern than that produced in human cells, and hence different protein stability. To determine whether the trend of thermal stability change of the wild-type and MSPβ R689C remain the same upon changes in glycosylation, the proteins were deglycosylated and their thermal stabilities were examined in addition to those of the glycosylated proteins (Fig. 1). The T_m_ values (the midpoint of the melting curves) were calculated as the maximum of the first derivative of the melting curve (Fig. 1 A).

The melting curves of the glycosylated proteins reveal a clear decrease in the T_m_ value of the mutant protein compared with wild-type MSPβ (ΔT_m_ = 1.6 K, Table 2). The glycosylation status of MSPβ strongly influences the thermal stability of the protein. The melting curves of both deglycosylated wild type and R689C MSPβ show a striking decrease in the T_m_ values compared with their glycosylated counterparts (ΔT_m_ = 3.8 K for wild-type and 4.7 K for R689C MSPβ, Table 1). The destabilizing effect of the R689C mutation is more pronounced with the deglycosylated MSPβs (ΔT_m_ = 2.5 K) than it is with the glycosylated proteins.

**Table 2:** Melting temperatures of wild-type and R689C MSP β-domain.

Protein	T_m_ (°C)	Standard deviation^a^ (°C)
WT: glycosylated	71.9	0.5
R689C: glycosylated	70.3	0.1
R689C dimer: glycosylated	68.7	0.3
WT: deglycosylated	68.1	0.1
R689C: deglycosylated	65.6	0.2

a The standard deviations were calculated from four independent melting curves.

Together, the DSF data show that MSPβ R689C is destabilized compared with wild type MSPβ. However, the stability decrease is likely too small an effect to have a significant impact in *in vivo* protein function. Examination of data for proteins where both ΔT_m_ and ΔΔG data are available, such as barnase [23], subtilisin [24], Plasminogen activator kringle domain [25], and T4 lysozyme [26], shows a 1 kcal mol^−1^ change in free energy difference between the folded and unfolded states typically corresponds to a 3 to 4 K change in melting temperature. On this basis, for glycosylated MSP, the destabilization is probably in the range 0.5 to 0.8 kcal mol^−1^. In contrast to this, monogenic disease causing mutations that act in a manner consistent with destabilization typically have a loss of stability of at least 2 to 3 kcal mol^−1^ [4]. The simplest model of the relationship between loss of stability and lower effective *in vivo* protein activity through increased concentration of the unfolded species supports an exponential relationship between the amount of destabilization and the expected change in concentration of unfolded protein, such that whereas a typical monogenic disease causing mutation increases the concentration of unfolded protein by approximately 100 fold, the destabilization resulting from this SNP is expected to cause an increase of approximately 3 fold. Nevertheless, the MSP variant may still have a modest effect on *in vivo* protein concentration.

### R689C MSPβ free cysteine assignment

As explained in Methods, TPS chromatography coupled with trypsin digestion and mass-spectrometry analyses was employed to identify free cysteines in MSPβ R689C. The recombinant MSPβ was engineered following the strategy employed for the crystal structure determination, including 19 amino acids of the linker to the α-chain, to enable formation of the physiologically relevant five disulfide bonds: Cys468-Cys588, Cys507-Cys523, Cys527-Cys562, Cys602-Cys667, Cys657-Cys685. Another cysteine, Cys672, is located in close proximity to the Cys468-Cys588 disulfide bond connecting the α and β chains. To avoid an aberrant disulfide bond, Cys672 was mutated into Ser. Thus, if all disulfide bonds formed correctly in the R689C MSPβ mutant, only the peptide that includes Cys689 would be detected. Trypsin cleaves peptides primarily after arginine residues. The arginine-flanked peptide sequence that spans Cys689 is: Arg687-Ser688-Cys689-Trp690-Pro691-Ala692-Val793-Phe694-Thr-695-Arg696. Only a single major peptide of R689C MSPβ was eluted from the TPS column. The peptide was sequenced by mass spectrometry as: Ser-Cys-Trp-Ala-Val-Phe-Thr-Arg, confirming that Cys689 did not form an aberrant disulphide bond. Thus, there is no evidence of an incorrect intramolecular disulfide bond.

### Analysis of MSPβ-RON interaction by SPR

**Figure 2:**
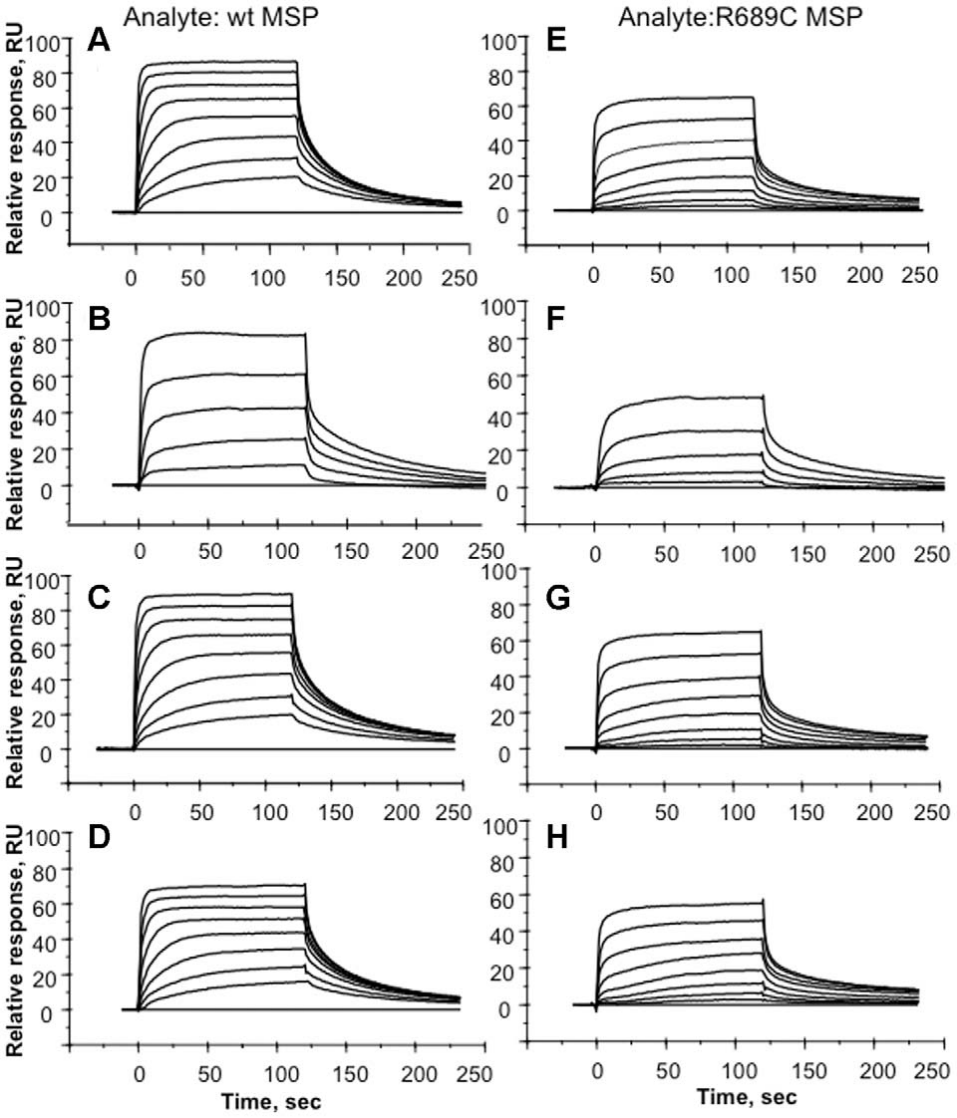
Sensorgrams of wild-type and R689C MSPβ binding to immobilized RON extracellular domain constructs. The binding curves in each sensogram correspond to increasing concentrations of the analyte in 2-fold dilution steps: 4, 8, 16, 32, 64, 128, 256, and 512 nM. The eight experiments correspond to immobilized: (A,E) RON Sema. (B,F) RON Sema-PSI. (C,G) RON Sema-PSI-IPT_1_. (D,H) RON Sema-PSI-IPT_1–4_. The response units (RU) were corrected for nonspecific binding relative to a blank flow cell surface.

**Figure 3:**
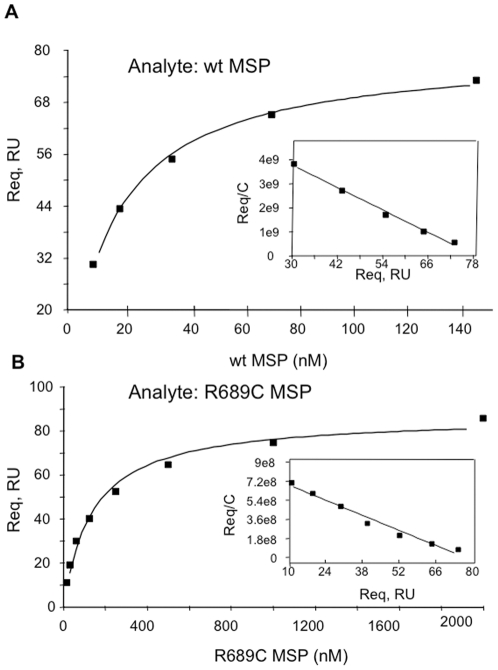
SPR equilibrium analysis of binding to immobilized binding to immobilized RON-Sema. (A) Wild-type and (B) R689C MSPβ. The inserts show the Scatchard plots.

Binding of the wild type and R689C MSPβ (including 19 amino acid of the MSP α-chain) to four immobilized extracellular variants of RON were measured by Surface Plasmon Resonance (SPR). The SPR data complement previous co-immunoprecipitation studies that concluded that MSPβ binds to the RON Sema domain [18]. In addition, the current study define *K*_d_ values under the SPR experimental conditions for both wild type and mutant MSPβ. Representative binding sensorgrams are shown in Figure 2. The curves show that both wild type and R689C MSPs bind to RON Sema, Sema-PSI, Sema-PSI-IPT_1_, and Sema-PSI-IPT_1–4_. The binding curves were fitted globally to a 1∶1 Langmuir kinetic model (Table 3), yielding a 10–13 fold tighter binding for the wild-type MSPβ (13.5–21.2 nM) compared with the mutant MSPβ (148–233 nM). Because the on and off binding rates were quite high, *K*_d_ values were also calculated using an equilibrium model (Fig. 3). Consistent with the kinetic model, the equilibrium model yielded a 7–8 fold tighter binding for the wild-type MSPβ (16.4–32.0 nM) compared with the MSPβ R689C (139–244 nM). In addition, the binding data show that the protein-protein interaction may be attributed primarily to the RON Sema domain alone with no significant contribution of the PSI and the four IPT domains, consistent with the reported 90% inhibition of MSPβ binding to immobilized full length RON by Sema or Sema-PSI using competitive ELISA experiments [18].

**Table 3:** SPR binding kinetics of wild-type and R689C MSP β-chain to the RON domains.

RON	Analyte	*k*_on_(M^−1^· s^−1^)×10^5^	*k*_off_(s^−1^)×10^−2^	*K*_d_ (nM)	*K*_d_ (nM)
construct^a^				1∶1 Langmuir	Equilibrium model
S-RON	wt MSP	23.1	3.1	13.3	16.4
SP-RON		13.3	2.8	21.2	32.0
SPI-RON		17.6	2.7	15.1	17.8
SPI_4_-RON		16.9	2.3	13.5	22.6
S-RON	R689C MSP	4.8	8.1	170.0	139.0
SP-RON		2.7	6.2	233.1	244.0
SPI-RON		8.7	12.9	148.0	154.0
SPI_4_-RON		3.5	5.5	157.0	158.0

a abbreviations: S – Sema, SP – Sema-PSI, SPI – Sema-PSI-IPT_1_, SPI_4_ – Sema-PSI-IPT_1–4_.

The SPR MSPβ/RON binding affinity is approximately 100-fold tighter than that between the homologous MET Sema-PSI and HGFβ as measured using the same SPR technique [27]. In contrast, the MET Sema-PSI SPR studies showed 10-fold tighter binding affinity to a HGFα construct than to HGFβ. An analogous SPR study of MSPα binding to RON domains has not been reported; however, ELISA studies suggested that MSPα binding affinity for RON is over 50-fold weaker than that of MSPβ [15]. The apparent low contribution of MSPα to RON binding implies that the 10-fold reduced RON binding affinity of MSPβ R689C cannot be masked by the RON/MSPα interaction.

**Figure 4:**
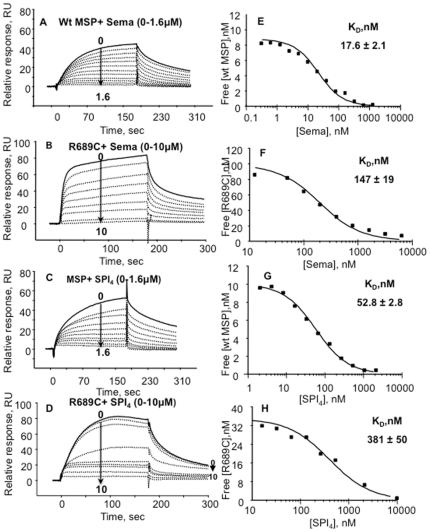
SPR competition assays. (A–D) Bindings to immobilized RON-Sema-PSI-IPT_4_ of mixtures containing MSPβ with varying concentrations of RON-Sema or RON-Sema-PSI-IPT_4_. (E–H) The dependence of free MSPβ concentrations on RON concentration in the solution mixtures. The free MSPβ concentrations were determined from the measured relative response based on a calibration curve.

To further examine the RON/MSPβ interaction, competition SPR [28] experiments measured capture of monomeric MSPβ from pre-equilibrated Sema/MSPβ and Sema-PSI-IPT_1–4_/MSPβ analyte mixtures to immobilized RON Sema-PSI-IPT_1–4_ (Fig. 4). The results confirmed that the Sema domain alone competes with the RON Sema-PSI-IPT_1–4_ for MSPβ binding. The calculated dissociation constants for wild type and R689C MSPβ (Table 3) were consistent with those calculated from the direct SPR binding experiments (Table 3).

Together, the SPR binding experiments show that the R689C MSPβ exhibits approximately 10-fold lower binding affinity to its receptor compared with the wild type MSPβ. The reduced affinity might impair the biological function of the MSP/RON signaling system.

### Structural consequences of the R689C mutation

**Figure 5:**
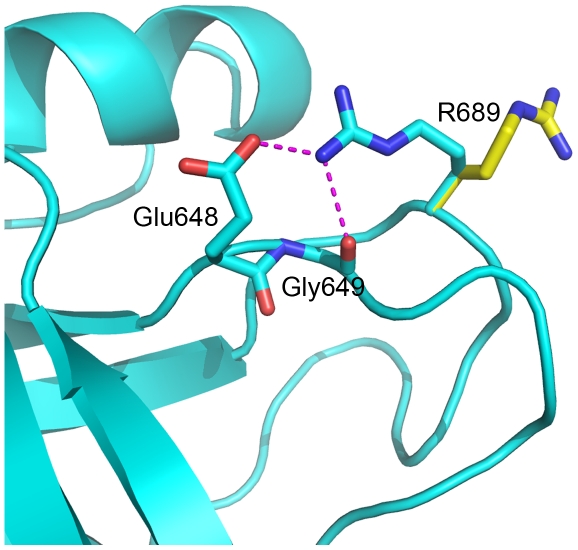
Side chain conformation of Arg689 in the MSPβ crystal and modeled structures. In the crystal structure (yellow), the side chain is involved in inter molecular crystal contacts, and in the modeled conformation (cyan) it forms an intra molecular salt bridge with Glu648 and a hydrogen bond with Gly649 backbone carbonyl. These interactions, if present or partly present in solution, may contribute the modest change in proteins stability introduced by the R689C substitution.

The crystal structure of MSPβ shows that Arg689 is located on a surface loop, fully exposed to solvent [22]. However, a symmetry-related molecule packs close to Arg689 in the crystals such that the side chain of Arg689 stacks against the side chain of Trp593 on a neighboring molecule, generating an energetically favorable π-π interaction that contributes to crystal stability. MSPβ exists as monomers in solution, thus such an inter-molecular interaction is unlikely. Instead, the structure reveals that Arg689 side-chain orientation may readily adjust to form an intra-molecular salt bridge between the Arg689 and Glu648 side chains and concomitantly form a hydrogen bond between the guanidinium group and the backbone carbonyl of Gly649 (Fig. 5). Both Glu648 and Gly649 reside on a loop adjacent to the Arg689 loop, thus these interactions may contribute to protein stability. In contrast, a cysteine at position 689 cannot form analogous interactions, which may explain the modest reduction in protein stability associated with the R689C MSPβ.

**Figure 6:**
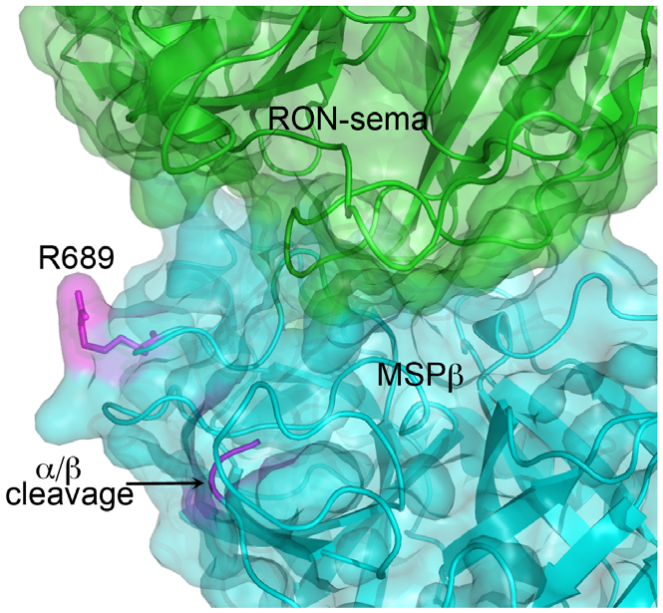
Model of the structure of MSPβ/RON Sema complex. Arg689 and the location of the α/β cleavage site are highlighted in magenta color. The molecular surfaces are shown in transparent colors. The model was built by super positioning the crystal structures of MSPβ [22] (PDB entry 1ASU) and RON-Sema-PSI (Chao and Herzberg, unpublished) on the crystal structure of the HGFβ/MET-Sema-PSI complex [29] (PDB entry 1SHY). In this model, Arg689 is not directly involved in the RON/MSP interface.

A model of the MSPβ/RON-Sema complex was generated using the structures of the individual proteins super positioned on the HGFβ/MET-Sema [29], as described in the methods section. This model places Arg689 outside the protein-protein interface, with the guanidinium group minimally 11 Å away from the carboxylate of Glu149 on RON Sema, the closest residue on the receptor (Fig. 6). Based on the model, substitutions at this position are unlikely to affect protein-protein affinity. Yet, the SPR studies showed that the R689C MSPβ exhibited approximately 10-fold reduced affinity towards RON constructs compared with the wild-type MSPβ.

A site directed mutagenesis study of a different MSP variant, R683Q, found no MSP/RON binding [15]. The MSPβ/RON-Sema model places Arg683 at the protein-protein interface, by analogy with HGFβ's Arg695. However, whereas the HGF Arg695 interacts with a MET tyrosine residue (Tyr125), the tyrosine counterpart on RON is an alanine residue (Ala128), which cannot participate in a guanidinium-aromatic interaction. Indeed, many of the interface residues are not conserved between MET Sema and Ron Sema or between HGFβ and MSPβ, consistent with the mutual exclusivity of the RON/MSP and MET/HGF pairing specificities [30]. Together, the SPR binding studies and the mutagenesis information support the notion that the protein-protein interfaces of the two systems resemble but are not identical to one another. Therefore, accurate mapping of the relationship between the Arg689 mutation site and the MSPβ/RON Sema interface requires the structure determination of the MSPβ/RON Sema complex.

### MSP activation

**Figure 7:**
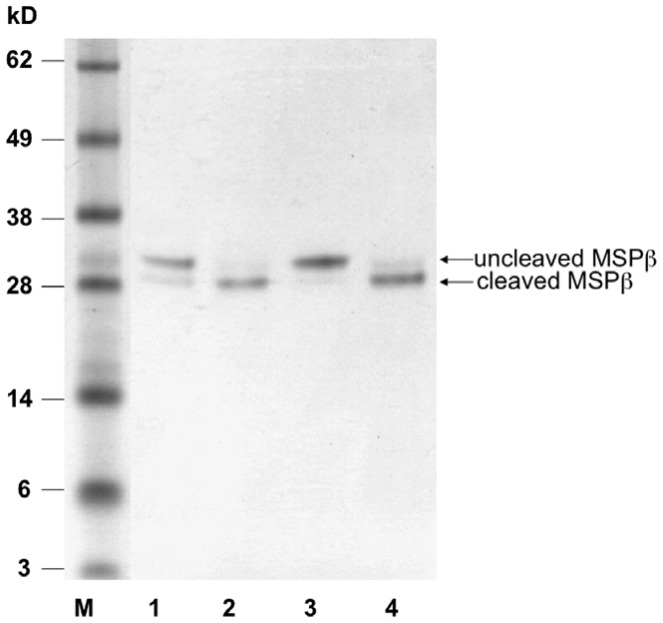
MSPβ SDS-PAGE performed at reducing conditions to assess cleavage by matriptase-1. Samples of MSPβ fused to the 19 C-terminal amino acids of MSPα before and after 4 hrs incubation with matriptase-1 at 37°C were applied. Lane 1 – wild type MSPβ; Lane 2 – wild type MSPβ after incubation with matriptase-1; Lane 3 – R689C MSPβ; Lane 4 – R689C MSPβ after incubation with matriptase-1; M – molecular weight markers.

The extracellular serine protease domain of the macrophage transmembrane protease, matriptase-1, activates pro-MSP [13] by specifically cleaving the α-β single polypeptide chain between Arg483 and Val484 to generate α and β chains that are linked by a single disulfide bond between Cys468 and Cys588. The Arg483-Val484 peptide has been cleaved in the crystal structure and Val484 is buried in the protein core. Because the exact position of the Arg483-Val484 peptide on the protein surface prior to cleavage is unknown, the adjacent Gly486, positioned on the MSPβ surface, is also highlighted in Figure 6. This illustrates that Arg689 is located remotely from the activation site, and thus the substitution by a cysteine is not likely to impair the peptide bond cleavage directly. Nevertheless, the transient MSP/matriptase interface formed during proteolysis might span large surface area and engage Arg689 such that the R689C substitution might interfere with this interaction and compromise MSP activation. Therefore, we investigated the cleavage by matriptase-1 of both wild type and mutant MSPβ, both of which contain the fused 19 C-terminal amino acid of MSPα. The cleaved and uncleaved proteins migrate the same distance on a SDS-PAGE gel under non-reducing conditions. In contrast, SDS-PAGE analysis under reducing conditions breaks the disulfide bonds and discriminates between the uncleaved β-chain and the shorter cleaved β chain that now lacks the MSPα 19 amino acid fragment. The experiment shows that incubation of the wild type and R689C MSPβ variants with matriptase-1 as described in the methods section followed by SDS-PAGE analysis under reducing conditions yields cleaved wild-type and mutant proteins (Fig. 7). Thus, the substitution does not interfere with MSP activation.

### Disulfide bridge formation and intramolecular interactions

Finally, there are two other potential consequences of the R689C mutation. First, as MSP is an extracellular protein, the free cysteine might form an inter molecular disulfide bond to generate MSP dimers that could interfere with binding to RON. SDS-PAGE under non-reducing conditions of a sample that was stored at 4°C for over six months revealed small amounts of MSPβ dimers. This dimeric protein was purified by gel filtration chromatography and its thermal stability was shown by DSF to be 1.5 K lower than that of the monomeric MSPβ R689C (T_m_ = 68.7°C). The dimeric MSPβ binding affinities towards RON constructs were unimpaired and were actually comparable with those of wild type MSPβ (*K*_d_ = 3.7–4.9 nM), perhaps owing to avidity effects. Investigation of the presence of MSP dimers in individuals carrying the R689C mutation would be required to determine whether dimerization plays a role *in vivo*. Nevertheless, these results suggest that even if dimers were formed, RON binding would not be impaired.

Second, it remains unknown whether MSPβ interacts closely with any of the α chain domains, and which of the MSP domains mediates the physiological dimerization of RON. If the Arg689 plays a role in such interactions, the R689C mutation might have a negative effect on function. Studies of the entire activated MSP containing both the α and β chain and its effect on RON dimerization would be required to shed light on these issues. Clearly, the cellular system is more complex than that studied *in vitro*; nevertheless, the different properties of the mutant MSPβ compared with the wild type MSPβ are significant and might contribute to the impaired biological function.

### Conclusions

Computational analysis of the GWAS results for the chromosome 3 Crohn's disease associated locus identifies the SNP resulting in the MSPβ R689C amino acid substitution as the only known viable missense candidate for involvement in disease mechanism in the locus. Computational impact analysis provides evidence that R689C is expected to have a significant effect on the *in vivo* function of the protein through some mechanism other than thermodynamic destabilization. Further support for the involvement of that SNP comes from its association with disease risk in a targeted gene study [7]. These results alone provide suggestive evidence for a role of the SNP in the disease, but the substitution might have an insignificant effect on *in vivo* function; or a mechanism other than missense, such as an effect on expression or translation, could be involved in this locus; or an unknown genetic variant in LD with the marker might be responsible. A number of possible functional impacts at the protein level have been examined. The results show that the substitution does not lead to non-native disulfide bond formation or impairment of the activation by matriptase-1. There is also a modest reduction in protein stability that might play a minor role. The major finding is that the substitution reduces the binding affinity to the RON receptor tyrosine kinase by an amount likely to seriously affect signaling: Assuming the *in vivo* concentration of RON and MSP are well below the *K*_d_ value of the complex, the lower affinity will result in an order of magnitude lower concentration of the signaling complex, likely reducing the normal function of the MSP/RON signaling pathway substantially. This in turn is likely to alter the inflammatory response, particularly the level of macrophage activity. Some Crohn's patients exhibit lower than normal macrophage response, providing further circumstantial evidence for such a mechanism.

## Materials and Methods

### Identification of candidate non-synonymous SNPs

As noted earlier, association studies identify those SNPs included in a microarray (tag SNPs) that are associated with an increased risk of disease. A marker may itself be involved in disease mechanism, or more probably, is in LD with one or more mechanism SNPs. Thus, given a set of marker SNPs in a susceptibility locus, we require the set of possible mechanism candidates. The availability of linkage disequilibrium data in Hapmap [31] provides a basis for obtaining such a list. A common approach is to extract all SNPs in strong LD with one or more markers in a locus from Hapmap LD information. This method omits viable candidates less strongly linked to the markers. A more rigorous approach is to impute the likely genotypes in each individual in the study for each Hapmap SNP not included in the microarray [32]. Since the genotypes of SNPs in weak LD with the markers cannot be imputed with certainty, the resulting probability of altered SNP frequency is underestimated. Imputation also requires the full data for individuals to be included in the study, which is not always easily available. We have introduced an alternative approach, designed to be easily applied to any dataset, and to produce an inclusive set of candidates. The method selects as candidates all SNPs for which the penetrance (the probability of having the disease, given the presence of a particular SNP) is as high or higher than that of the marker SNPs in a locus. That is:
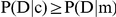
where P(D|c) is the probability of an individual having the disease, given they carry the candidate allele ‘c’, and P(D|m) is the probability of an individual having the disease, given they carry the marker allele. Penetrances are calculated from Hapmap data [31] and the population frequencies of the marker and candidate SNPs. In addition, any SNP for which the linkage disequilibrium correlation r^2^> = 0.8 is included.

### Identification of high impact non-synonymous candidate SNPs

Identification of SNPs that significantly destabilize the folded structure of a protein molecule [4]. A set of structural effects, such as reduction in hydrophobic area, over-packing, backbone strain, and loss of electrostatic interactions, is used to represent the impact of single residue mutations on protein stability. A SVM was trained on a set of mutations causative of monogenic disease (extracted from an early version of the Human Gene Mutation Database [33]) and a control set of non-disease causing mutations. In jack-knifed testing, the method identifies 74% of disease mutations with a false positive rate of 15%. The apparent false negative rate includes both true false negatives (cases where stability is involved, and the method classification is incorrect) and cases where the contribution to disease arises from some factor other than stability. When only higher confidence assignments are included (those with an SVM score <−0.5), the false positive rate is 11%. Use of the method to evaluate a set of *in-vitro* mutagenesis data with the SVM established that the majority of monogenic disease mutations affect protein stability by 1 to 3 kcal mol^−1^ [4].

Identification of high impact SNPs through amino acid sequence conservation properties: The extent of family sequence conservation and types of residue observed at a non-synonymous SNP position are considered. The more restricted the amino acid, the more likely that a different or unusual residue at that position will be deleterious to protein function. A SVM is also used to identify high impact substitutions in this model, using the same disease and control datasets as for the stability method. In jack-knifed testing, the method identifies 80% of disease mutations with a false positive rate of 10% (for high confidence assignments, 16% and 6% false negative and false positive rates, respectively). This method has the advantage that it does not require knowledge of structure and so can be applied to a larger fraction of SNPs. It has the disadvantage that it provides no direct insight into the nature of the impact on protein function. (See [5] for a full description).

### Production and purification of recombinant RON extracellular domain constructs

The extracellular region of the human MST1R gene was amplified from the pMSCVneo-hRON-2HA clone (kindly provided by Dr. Pamela A. Hankey, Penn State University) and was ligated into a BglII/AgeI digested pMT/BiP/V5-HisA vector for secreted expression as a C-terminal non-cleavable His_6_-tagged recombinant protein in *Drosophilae melanogaster* Schneider 2 (S2) cells (Drosophila Expression System, Invitrogen). The putative furin cleavage site in the RON Sema domain (Lys305-Arg306-Arg307-Arg308-Arg309-Gly310-Ala311) was mutated to a thrombin (Lys305-Leu306-Val307-Pro308-Arg309-Gly310-Ser311) or a TEV protease (Glu304-Lys305-Arg306-Tyr307-Arg308-Gln309-Gly310) cleavage site. The recombinant proteins contain two residues (Arg23 and Ser24) at the N-terminus and eight amino acids (Thr684, Gly685, and His686-His691) at the C-terminus, derived from the expression vector. Four different RON constructs were engineered: Sema (Glu25-Gly524), Sema-PSI (Glu25-Pro568), Sema-PSI-IPT_1_ (Glu25-Glu683), and Sema-PSI-IPT_1–4_ (Glu25-Ser956). The sequences of all expression clones were confirmed by DNA sequencing.

*Drosophilae melanogaster* S2 cells were transfected with the RON expression vectors and pCoPuro, and stable transfectants resistant to puromycin were selected. The amount of protein in the conditioned serum free media (HyClone SFX) was determined by Western blot analysis with the Penta-His monoclonal Antibody (Qiagen) and clonal selection of stable transfectants was conducted to obtain clones with improved expression levels. For large-scale expression, stable S2 cells were grown in shaker flasks at 28°C and protein expression from the metallothionin promoter was induced by the addition of 0.6 mM CuSO_4_. The cells were removed by centrifugation ∼4–5 days post induction and the cell-free conditioned medium was applied directly onto a Chelating Sparse Fast Flow column, pre-equilibrated in MilliQ water (GE Health Sciences) [34]. The resin was washed with phosphate buffered saline followed by 0.5 M NaCl to remove nonspecifically bound proteins and contaminants. The bound protein was eluted with 50 mM Tris-HCl, pH 8.0, containing 50–500 mM Imidazole and fractionated sequentially with 45% and 80% ammonium sulfate. The eluted protein was purified using the Sephacryl S200 size exclusion chromatography (GE Health Sciences) in 20 mM Tris-HCl, pH 7.8, 0.10 M NaCl, 0.5 mM ethylene-diamine-tetra-acetic acid (EDTA). Purification of the RON Sema-PSI-IPT1_1–4_ required an additional ion-exchange chromatographic step using Source 30Q.

Protein purity and identity were monitored by sodium dodecyl sulfate-polyacrylamide gel electrophoresis (SDS-PAGE) and western analysis using the Penta-His monoclonal antibody (Qiagen). The concentration of RON Sema, Sema-PSI, Sema-PSI-IPT_1_ and Sema-PSI-IPT1_1–4_ were determined using the extinction coefficients of 27,235 M^−1^ cm^−1^, 42,745 M^−1^ cm^−1^, 44,485 M^−1^ cm^−1^, and 67,695 M^−1^ cm^−1^, respectively. The protein yields per liter of conditioned medium were ∼1 mg, ∼1.5 mg, ∼2.5 mg, and 0.2 mg, respectively. Size exclusion chromatography and MiniDawn TREOS multiangle light scattering (Wyatt Technology) indicated that the protein was monomeric. Matrix-assisted laser desorption time-of-flight (MALDI-TOF) spectrometric analysis of the RON recombinant proteins gave MW = 56,444±313 Da for Sema (∼Δ1957 Da greater than the calculated MW = 54,487 Da), 64,039±162 Da for Sema-PSI (∼Δ4710 Da greater than the calculated MW = 59,329 Da), 77,689±2 35 Da for Sema-PSI-IPT_1_ (∼Δ5789 Da greater than the calculated MW = 71,908 Da) and 108,971±366 Da for IPT1_1–4_. (∼Δ8308 Da greater than the calculated MW = 100,663 Da). The higher molecular masses are attributed to post-translational modifications. The NetNGlyc web server predicts 8 potential N-glycosylation sites for the amino acid sequence Asn-Xaa-Ser/Thr, four on the Sema domain and one on each IPT domain (http://www.cbs.dtu.dk/services/NetNGlyc/).

### Production and purification of recombinant wild type and R689C MSPβ

The recombinant human MSPβ was contracted from the MSP cDNA (clone ID: 5190966, Open Biosystems) following the design strategy employed for the protein production that yielded the crystal structure [22] except that the protein for the current study was produced in S2 cells. The recombinant MSPβ was cloned into AvaI/PmeI sites of the pMT/BiP/V5-HisC vector (Invitrogen). It includes the adjoining N-terminal 19 amino acid residues of the α-chain (residues Phe465-Gly711). Cys672 was mutated to a serine (Quick Change™_,_ Stratagene) to prevent formation of a wrong disulphide bond owing to the unpaired Cys672, which is located close to two other cysteine residues that form a disulfide bond [22]. This variant will be referred to as the wild type protein. The mutation, R689C, associated with CD and UC was engineered as well. The wild type and mutant MSPβ constructs were confirmed by DNA sequencing.

Drosophila S2 cells (Invitrogen) were transfected with the MSPβ wild type or R689C expression vectors and pCoBlast, and stable transfectants resistant to blasticidin were selected. For protein production, stable transfectants were grown in SFX medium supplemented with 20 mM L-glutamine. Protein expression was induced with 0.5 mM CuSO4. The cells were removed by centrifugation 4 days post induction. The conditioned medium was consecutively passed through Chelex 100 (BioRad) and QSepharose FF (GE Health Sciences) resins to remove Cu^2+^, DNA, and some contaminating proteins. The QSepharose flow-through fraction was concentrated with QuixStand Cross flow concentrator (Amersham Biosciences) and applied on Talon Co^2+^-chelated Sepharose resin (Clontech), equilibrated with 50 mM potassium phosphate (pH 7.5), 0.5 M NaCl and 10 mM Imidazole. The His-tagged MSPβ was eluted by the above buffer containing 150 mM Imidazole and further purified on Superdex 200 HR column (GE Health Sciences) equilibrated in 10 mM Tris-HCl (pH 8.0), and 100 mM NaCl. Size exclusion chromatography and multiangle light scattering showed that the protein existed in solution as monomers.

Protein concentrations were determined by using molar extinction coefficient at 280 nm of 50,920 M^−1^ cm^−1^. The purification yielded ∼2 mg MSPβ from 1 liter of conditioned medium. MALDI-TOF spectrometric analysis of the wild type and R689C MSPβ recombinant proteins gave MW = 29,260±33 Da (∼Δ916 Da greater than the calculated MW = 28,344) and 29,370±42 Da (∼Δ1096 Da greater than the calculated MW = 28,274), respectively. MSPβ has a single potential N-glycosylation site at Asn615. Thus, the difference in molecular mass is consistent with post-translational modification at a single glycosylation site containing 6–7 sugar units.

Both wild type and mutant MSPβ were treated with the glycoamidase PNGase F to removed the N-glycosyl group. The recombinant PNGase F from *Elizabethkingia miricola* (ATTC 33958D) was produced in house as described previously [35]. A solution containing 100∶1 ratio of MSPβ and PNGase F in 50 mM potassium phosphate buffer (pH 7.5) was incubated for 3 h at 37°C. The deglycosylated MSPβ was purified by size-exclusion chromatography on Superdex 200 HR column in 20 mM MES buffer (pH 6.0) and 100 mM NaCl. The MALDI-TOF analyses yielded MW = 28,260±18 Da and MW = 28,362±22 Da for the wild type and R689C MSPβ, respectively, consistent with the theoretical molecular mass of the non-glycosylated proteins.

### Differential Scanning Fluorimetry

The DSF experiments were performed using a Mastercycler ep realplex quantitative real-time (RT) PCR system (Eppendorf, Hamburg, Germany). The MSPβ samples were subjected to heating as described previously [36]. Briefly, protein stock solutions (0.5 mg/mL) in 10 mM Na^+^HEPES buffer (pH 7.3), 150 mM NaCl, were mixed with freshly diluted Sypro Orange (10×) (Invitrogen, Paisley, Scotland, U.K.) to generate 20 µL reaction solutions in a Fast Optical 96-well plate (Applied Biosystems). After sealing the plate with an optical adhesive film (Applied Biosystems) and centrifugation, the sample were monitored in the RT-PCR instrument as follows. The heating protocol comprised a pre-warming step at 30°C for 120 s and a subsequent heating gradient from 30 to 90°C at 2°C/min steps. Data were collected using a ROX dye (Applied Biosystems) calibration detection settings. The melting curves were obtained for the glycosylated and deglycosylated forms MSPβ wild type and R689C. The melting temperature (T_m_) of each melting curve was calculated as the maximum of the first derivative of the curve. The average T_m_ value of each protein was determined from four distinct melting curves.

### Free cysteine identification in R689C MSPβ

Thiopropyl Sepharose 6B (TPS) reacts with proteins containing free thiol groups under non-reducing conditions to form disulfide bonds, allowing the separation of peptides containing reduced thiol groups from those containing disulfide bonds. TPS chromatography coupled with trypsin digestion and mass-spectrometry analyses identified the free cysteine in MSPβ R689C as follows. MSPβ R689C, covalently bound to TPS beads (GE Life Sciences) in 50 mM NH_4_HCO_3_ buffer (pH 7.7), was treated with trypsin (Sigma-Aldrich) at 1∶25 (w/w) enzyme/substrtate ratio for 40 min at 37°C. Following proteolysis, the TPS beads were washed with buffer containing 50 mM NH_4_HCO_3_ (pH 7.7) and 150 mM NaCl to remove non-specifically bound cleavage fragments. The bound peptides were eluted from the column in the presence of 10 mM DTT. The eluted fractions were treated with 50 mM iodoacetamide for 60 min at room temperature. Small fraction aliquots were then mixed with equal volumes of 10 mg/mL α-cyano-4-hydroxycinnamic acid dissolved in 50% acetonitrile and 0.1% TFA, and spotted onto an ABI 01-192-6-AB target plate. Mass spectrometry (MS) analysis was performed using an AB4700 Proteomics Analyzer (Applied Biosystems, Framingham, MA, USA). MS-mode acquisitions consisted of 1,000 laser shots averaged over 20 sample positions. For MS/MS-mode acquisitions, 3,000 laser shots were averaged over 30 sample positions for post-source decay fragments. Automated combined acquisition of MS and MS/MS data was controlled with the 4000 Series Explorer software 3.0. Data analysis was performed with the GPS Explorer software 3.5 utilizing Mascot 2.0 (MatrixScience, London, UK) as the search engine. During searching, the mass tolerance was 0.08 Da for the precursor ions and 0.2 Da for the fragment ions. Because of the presence of the R689C mutation, data analysis was also performed manually using the monoisotopic masses of tryptic peptides predicted for MSPβ R689C by the PeptideMass software (http://ca.expasy.org/tools/peptide-mass.html).

### Surface Plasmon Resonance

Binding of wild type and R689C MSPβ to the various extracellular RON domain combinations was measured using a Biacore T100 (GE Health Sciences). Purified Sema, Sema-PSI, Sema-PSI-IPT_1_, and Sema-PSI-IPT_4_ were covalently coupled in a random orientation through their primary amine groups to the carboxymethylated dextran matrix of a CM5 sensor chip (Series S; GE Health Sciences) according to the manufacturer protocol. All proteins were diluted to a concentration of 15 µg/mL in 10 mM Na^+^Acetate (pH 4.0 for Sema and Sema-PSI or pH 5.0 for Sema-PSI-IPT_1_ and Sema-PSI-IPT_1–4_). Three RON variants were immobilized on three sensor chip flow cells with matrix volume concentration of 10–20 fM. For reference, the fourth flow cell was treated only with the surface-activating reagents N-hydroxysuccinimide/N-(3-dimethylaminopropyl)-N′-ethylcarbodiimide (NHS/EDC). After protein immobilization, the chip surfaces were blocked by 1 M ethanolamine (pH 8.5) and unbound proteins were removed by washing with 50 mM NaOH. Two sensor chips were required to immobilize the four RON variants, thus two of the variants were included twice. One chip included Sema, Sema-PSI-IPT_1_, and Sema-PSI-IPT_1–4_ and the second chip included Sema-PSI, Sema-PSI-IPT_1_, and Sema-PSI-IPT_1–4_.

Binding assays were performed in a running buffer comprising 10 mM Na^+^HEPES (pH 8.0), 150 mM NaCl, and 0.005% Surfactant P20 (GE Health Sciences) at 25°C. To reduce non-specific binding, the immobilized chips were equilibrated with the running buffer, supplemented with 0.2 mg/mL bovine serum albumin, at a flow rate of 10 µL/min for several hours, followed by priming with the running buffer alone. To characterize binding kinetics of the wild-type MSPβ, concentrations of the protein ranging from 5 to 500 nM, were prepared by step dilution and injected over the RON surfaces for 2 min at a 50 µL/min flow rate. Each injection was followed by a 2-min dissociation period. For the experiments with the MSPβ R689C, the concentrations ranged between 32 and 2000 nM. All injections were performed using the Wizard “Customized Application” program in an automated mode. At the end of each cycle, the sensor chip surfaces were regenerated by a 0.5 min injection of 25 mM NaOH followed by a 2 min wash with running buffer.

Competition SPR experiments probed the MSPβ/RON interaction further following a previously published approach [28]. The experiments measured the binding of free MSPβ (wild-type and R689C) from a pre-equilibrated RON Sema/MSPβ or Sema-PSI-IPT_1–4_/MSPβ analyte mixtures to covalently immobilized Sema-PSI-IPT_1–4_. MSPβ concentrations close to the calculated *K*_d_ values (10 nM for wild-type MSPβ or 35–100 nM for MSPβ R689C) were mixed with increasing concentrations (0.1–10,000 nM) of Sema- or Sema-PSI-IPT_1–4_ RON and pre-equilibrated for at least 30 minutes. The pre-equilibrated complexes were sequentially injected into the sensor chip to detect free MSPβ binding to the immobilized RON. The concentration of unbound MSPβ was calculated from calibration curves, where the initial binding rate of an analyte was plotted against the analyte concentration. For the calibration, the initial binding rates were determined from the set of binding sensorgrams for 1.5–12.5 nM (wild-type) or 4–62.5 nM (R689C) MSPβ to the immobilized Sema-PSI-IPT_1–4_. Each curve was fitted in the early association phase, defining linear correlation between the SPR signal and the analyte concentration. This calibration was used to determine the free MSPβ concentrations in the RON/MSPβ mixtures, which were then plotted against the soluble RON concentrations and globally fitted to a solution affinity model, yielding the affinity equilibrium constant (*K*_d_) in solution.

Kinetic data were analyzed using the BIAevaluation software, version 3.1 (BIAcore). All binding curves were corrected for background and bulk refractive index contribution using the reference flow cell and buffer alone. The sets of binding curves were globally fitted with a non-linear least squares algorithm, using single-exponential functions (Langmuir monovalent binding model). Kinetic parameters (*k*_on_ and *k*_off_) and equilibrium dissociation constants (*K*_d_) were determined based on at least two experiments.

### Proteolytic activation of MSP

The wild-type and R689C MSPβ proteins used in this study contain the C-terminal 19 amino acids of the MSPα fused to the N-terminus of the β-chain, including Cys468 of MSPα that forms an inter chain disulfide bond with Cys588 on the β chain. The specific proteolysis of the Arg483-Val484 peptide bond that activates MSP yields an α chain peptide that is linked to the β-chain only by the disulfide bond. Cleavage of both the wild type and R689C proteins by matriptase-1 was performed as follows. Samples containing 2 µL (500 nG) of MSPβ and 10 µL of 100 nM matriptase-1 (EnzoLife Sciences International, USA) were incubated for 4 h at 37°C at substrate to enzyme molecular ratio of 20∶1. Wild type and mutant MSPβ incubated in the absence of matriptase-1 served as controls. The proteolytic products were analyzed by SDS-PAGE (Invitrogen) under reduced conditions.

### Structure modeling

A comparative model of the MSPβ in complex with RON Sema-PSI was built based on the available crystal structures of MSPβ [22] (PDB entry 1ASU), RON Sema-PSI (Chao and Herzberg, unpublished), and the crystal structure of the homologous protein complex HGFβ/MET Sema-PSI [29] (PDB entry 1SHY) (HGFβ and MSPβ share 43% sequence identity, and the Sema-PSI domains of MET and RON share 29% sequence identity). The model was built by super positioning the individual protein structures on their respective homologs within the HGFβ/MET Sema-PSI complex, using the interactive computer graphics program COOT [37].
